# Author Correction: Mice adaptively generate choice variability in a deterministic task

**DOI:** 10.1038/s42003-020-0785-8

**Published:** 2020-01-31

**Authors:** Marwen Belkaid, Elise Bousseyrol, Romain Durand-de Cuttoli, Malou Dongelmans, Etienne K. Duranté, Tarek Ahmed Yahia, Steve Didienne, Bernadette Hanesse, Maxime Come, Alexandre Mourot, Jérémie Naudé, Olivier Sigaud, Philippe Faure

**Affiliations:** 1 Sorbonne Université, CNRS, Institut des Systèmes Intelligents et de Robotique (ISIR), 75005 Paris, France; 2 Sorbonne Université, INSERM, CNRS, Neuroscience Paris Seine - Institut de Biologie Paris Seine (NPS - IBPS), 75005 Paris, France

**Keywords:** Reward, Learning algorithms

Correction to: *Communications Biology* 10.1038/s42003-020-0759-x, published online 21 January 2020.

The original version of this Article contained an error in the spelling of the author Alexandre Mourot, which was incorrectly given as Alex Mourot.

This has now been corrected in both the PDF and HTML versions of the Article.

