# Supplementary material for: Mice adaptively generate choice variability in a deterministic task
Source: Commun Biol. 2020 Jan 21;3:34. doi: 10.1038/s42003-020-0759-x (PMC6972896; doi:10.1038/s42003-020-0759-x)
Supplement: Supplementary file 1 — Supplementary Information [file 42003_2020_759_MOESM1_ESM.pdf]

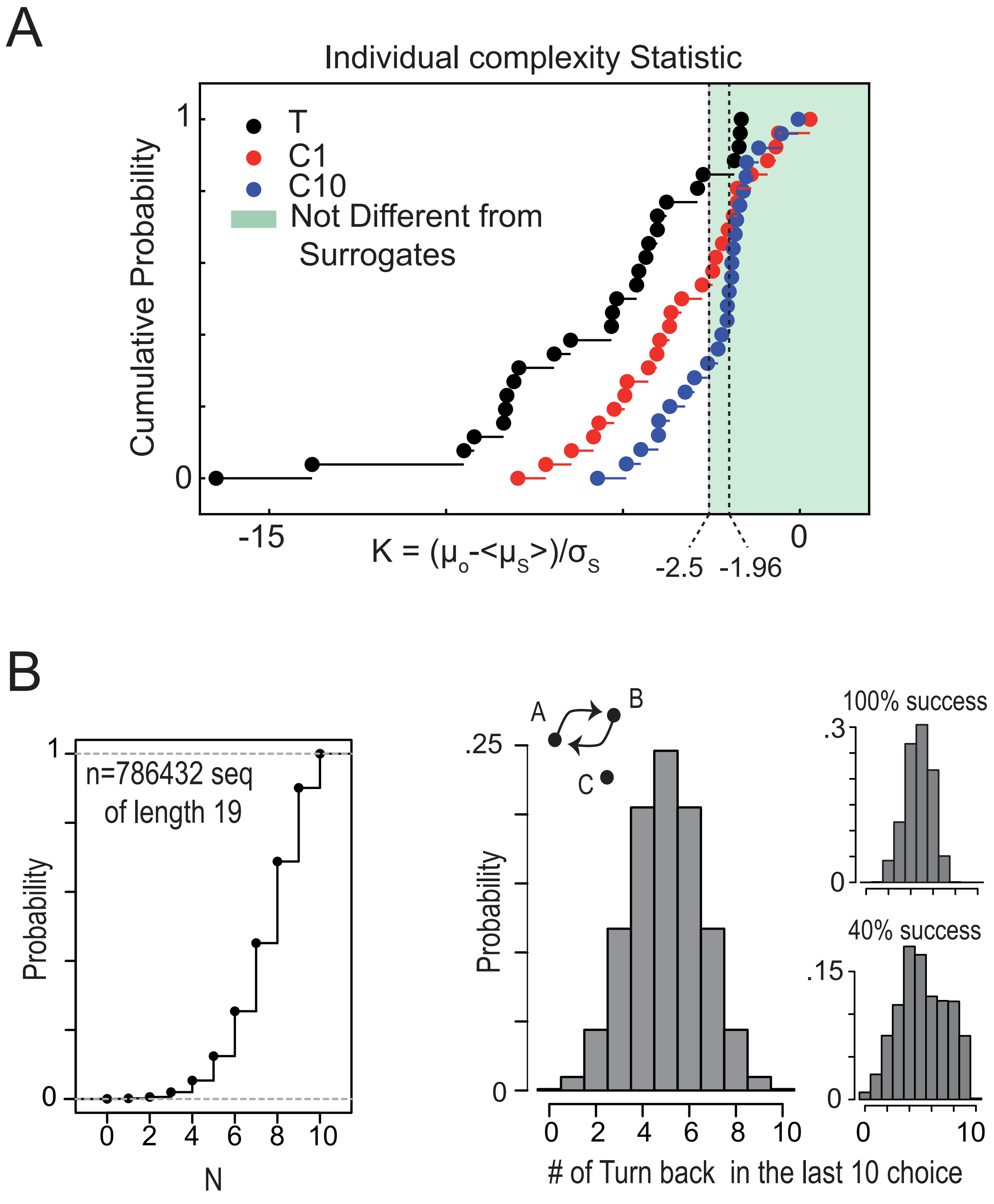

Supplementary Figure 1

Supplementary Figure 1: A) Individual comparison with random sequence of choices: Cumulative distribution of the K parameters calculated for each sequence of choices in sessions Training (T), C1 and C10. For each experimental sequence of choices, the term $K=(\mu_o-<\mu_s>)/\ \sigma_s$ was calculated, where μ_o is the complexity of the original data, $<\mu_s>$) and σ_s are the mean and standard deviation of the complexity of the surrogate series (i.e. for each experimental series, 1000 random sequences of the same length in which two consecutive elements could not be equal). We then tested the hypothesis that each original set was different from surrogates. Assuming Gaussian statistics, a limit of K=-2.5 and -1.96 indicates respectively a confidence of 99.4% and 95% that $\mu_o \geq \mu_s$. We found that 65.4% of mouse sequences in session C10 were not different from surrogates with a confidence of 99.4%. N = 27 in all sessions except C10 where N = 26. **B) Theoretical number of rewards and U-turns in the last 10 choices of a sequence of length 19:** *Left*: Cumulative distribution of the number of rewards obtained in the last 10 choices of the total number of sequences of length 19. Middle: Histogram of the number of U-turns in the last 10 choices. *Right*: Histogram of the number of U-turns in all possible sequences with 100% of reward (top) or 40% of reward (bottom). The optimal U-turn rate to maximize rewards is 50%.

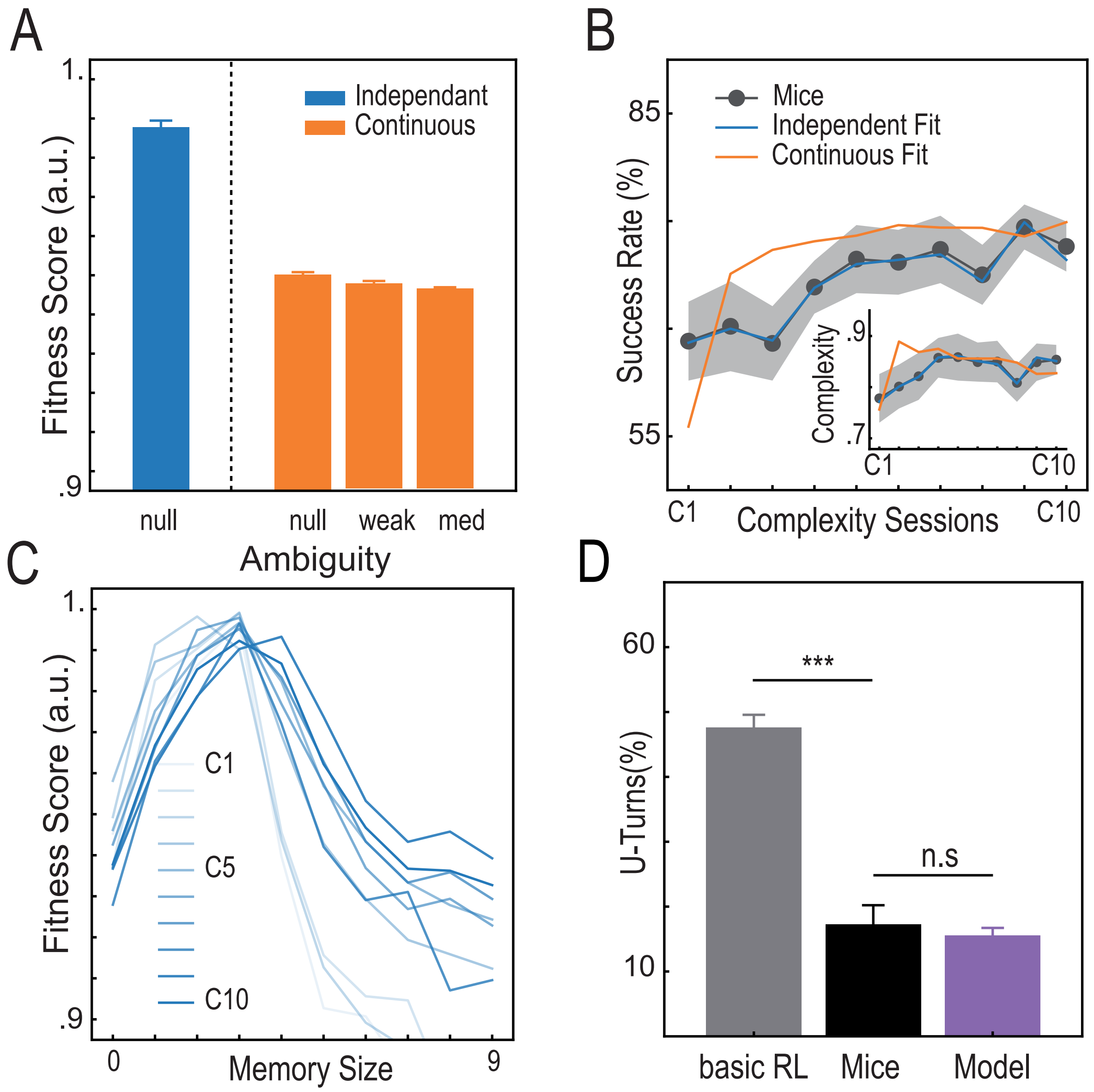

Supplementary Figure 2

Supplementary Figure 2: Model fitting results for different variants of the model. A), **B)** and **C)** Comparison of fitness scores with independent and continuous model fitting procedures. 'Independent' refers to the session-by-session hyperparameter optimization, 'continuous' to the optimization of the model for all sessions in a row (see 'Methods'). **A)** Comparison of the fitness score obtained by the N = 15 best fits in the independent and continuous fitting procedure. In continuous fitting, three levels of state representation ambiguity are shown (see 'Methods'). Error bars represent 95% confidence intervals. **B)** Success rates and complexity levels obtained in simulations in comparison with those obtained by mice. The shaded area represents the 95% confidence interval for mice. Only best fits are represented for model simulations (average of 20 runs). The model fitted for each session independently follows the evolution of behavioral data by adapting the exploration hyperparameter. By contrast, fitting all sessions with one single parameter set fails to reproduce the same evolution. In particular, the model exhibits a radical increase in success rate and complexity between sessions C01 and C02 as opposed to a more gradual progress in mice. **C)** Best fitness scores obtained with each of the tested memory sizes session by session. Smaller memories fit better. **D)** Comparison between the U-turn rates achieved by a basic RL algorithm, by our model and by mice in the training phase. A basic RL algorithm is unable to reproduce the stereotypical circular trajectories observed in mice during and after training; see also Figure 1B for mouse trajectories). Indeed, with equal probabilities of reward at all targets, this algorithm learns equal state-action values and randomly chooses between them. Discounting the reward function by a U-turn cost representing previous locations in the state vector (see 'Methods') makes the model capable to generate the same percentage of U-turns as mice under the training condition.

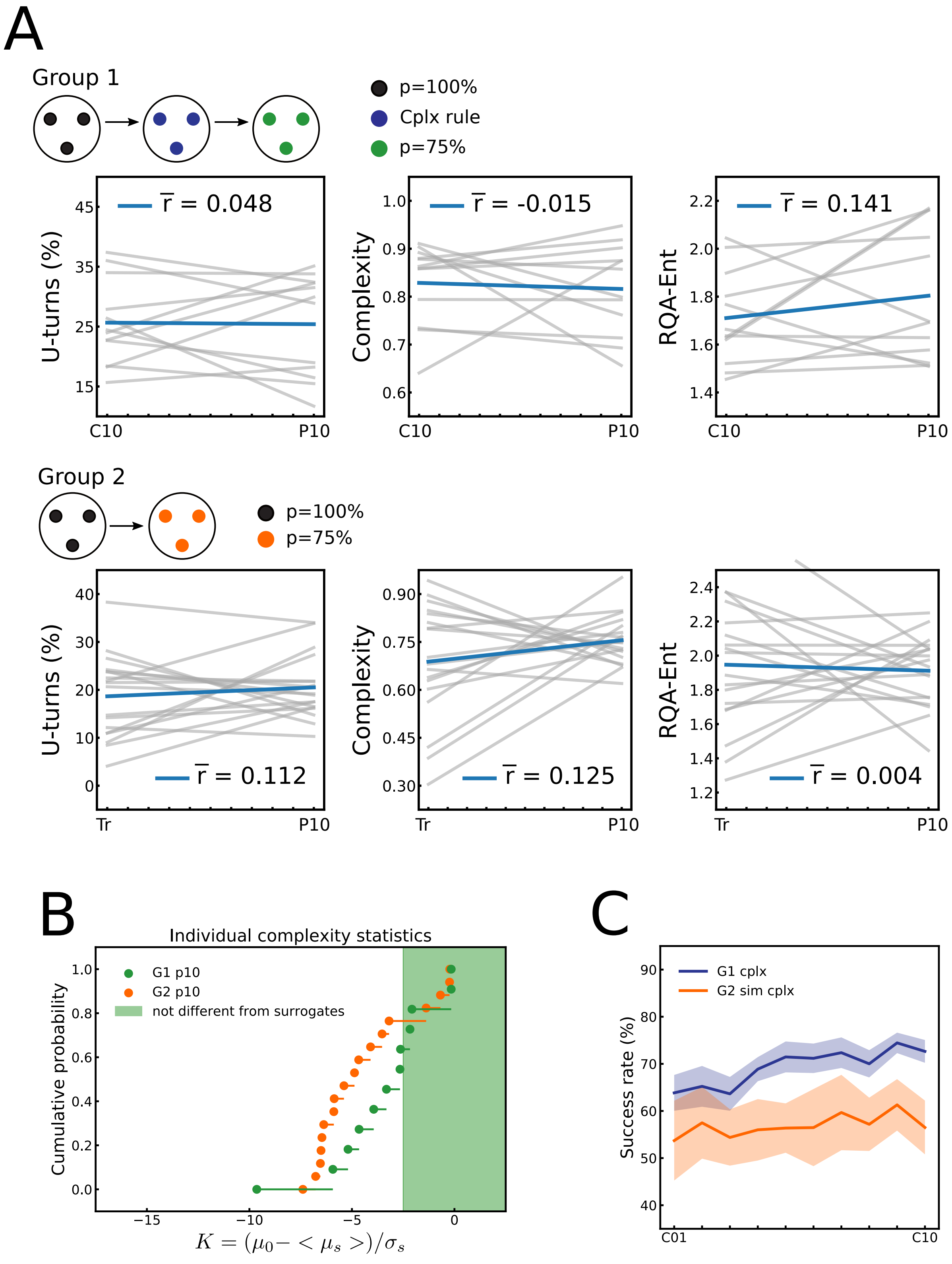

Supplementary Figure 3

Supplementary Figure 3: Absence of change in terms of U-turn rate, complexity and RQA entropy in the probabilistic condition compared to the previous condition. *Top*: Animals from Group 1. *Bottom*: Animals from Group 2. Grey lines represent linear regressions of the three measures for individual mice and the blue line represents the average. **B) Individual comparison with random sequence of choices:** Cumulative distribution of the K parameters calculated for each sequence of choices in the last sessions under the probabilistic rule p10 for G1 and G2 animals. For each experimental sequence of choices, the term $K=(\mu_o-<\mu_s>)/\ \sigma_s$ was calculated, where μ_o is the complexity of the original data, $<\mu_s>$) and σ_s are the mean and standard deviation of the complexity of the surrogate series (i.e. for each experimental series, 1000 random sequences of the same length in which two consecutive elements could not be equal). We then tested the hypothesis that each original set was different from surrogates. Assuming Gaussian statistics, a limit of K=-2.5 and -1.96 indicates respectively a confidence of 99.4% and 95% that $\mu_o \geq \mu_s$. We found that 33.3% of G1 mouse sequences and 22.3% of G2 mouse sequences were not different from surrogates with a confidence of 99.4%. N = 12 for G1, N = 18 for G2. **B) Success rate of the sequences performed by G2 animals if they were performed under the complexity rule:** We simulated the reward delivery rule of the complexity condition against the sequences performed by G2 mice under the probabilistic condition. Under these conditions, the animals would have reached a significantly lower success rate starting from the third session.

Supplementary Table 1: Hyperparameter ranges used in random search in independent, session-by-session model fitting ($N_{samples} = 6000$) and in grid search in continuous, all-sessions model fitting.

Hyperparameter random search ranges

**Label**	**Range**	**Step**	**Description**
m	$[0, 9]$	1	Memory size
τ	$[1, 20]$	*continuous*	Softmax temperature
κ	$[0, 1]$	*continuous*	U-turn cost

Hyperparameter grid search ranges

**Label**	**Range**	**Step**	**Description**
m	$[0, 9]$	1	Memory size (no ambiguity)
	$[0, 7]$	1	Memory size (low ambiguity)
	$[0, 5]$	1	Memory size (medium ambiguity)
α	$2^{-i}, i \in [0, 10]$	1	Learning rate
τ	$2^{i}, i \in [-4, 4]$	1	Softmax temperature
κ	$[0.5, 0.95]$	0.05	U-turn cost
