# Supplementary material for: Mice adaptively generate choice variability in a deterministic task
Source: Commun Biol. 2020 Jan 21;3:34. doi: 10.1038/s42003-020-0759-x (PMC6972896; doi:10.1038/s42003-020-0759-x)
Supplement: Supplementary file 3 — Peer Review File [file 42003_2020_759_MOESM3_ESM.pdf]

Editorial Note: *This manuscript has been previously reviewed at another Nature Research journal. The manuscript was considered suitable for publication without further review at Communications Biology.*
